# Upregulation of Multidrug Resistance-Associated Protein 1 by Allyl Isothiocyanate in Human Bronchial Epithelial Cell: Involvement of c-Jun N-Terminal Kinase Signaling Pathway

**DOI:** 10.1155/2015/903782

**Published:** 2015-07-27

**Authors:** Shujun Wang, Shanshan Wang, Chenyin Wang, Yajun Chen, Jie Li, Xueqi Wang, Dianlei Wang, Zegeng Li, Zhaoliang Peng, Ling Fan

**Affiliations:** ^1^Anhui University of Chinese Medicine, Hefei, Anhui 230038, China; ^2^People's Hospital of Feidong County, Hefei, Anhui 230001, China; ^3^Faculty of Health Sciences, University of Macau, Macau; ^4^The First Affiliated Hospital to Anhui University of Chinese Medicine, Hefei, Anhui 230031, China

## Abstract

Multidrug resistance-associated protein 1 (MRP1) plays a protective role in the etiology and progression of chronic obstructive pulmonary disease (COPD) which results from oxidative stress and inflammation of lung injury. The lower functional MRP1 activity is related to COPD development. Our previous study showed that Allyl isothiocyanate (AITC) induced the expression and activity of MRP1 in a dose-dependent manner. However, which signaling pathway contributes to the upregulation of MRP1 by AITC is unclear. In this study, signaling pathway specific inhibitors were used to examine the mechanism of AITC. We found that JNK inhibitor SP600125 treatment decreased MRP1 mRNA expression in 16HBE14o- cells. But the ERK inhibitor U0126 or PI3K/Akt inhibitor LY294002 produced no obvious effect. The AITC-induced increase of MRP1 mRNA expression was abolished by cotreatment of SP600125, while it was not obviously affected by U0126 or LY294002. Furthermore, AITC acivates the JNK signaling pathway in 16HBE14o- cells. Finally, we found that JNK pathway mediated the upregulation of AITC-induced expression and function of MRP1. Taken together, our results indicated that AITC increased the expression and the activity of MRP1 via a JNK-dependent pathway. ERK and PI3K signaling pathway were not involved in the expression of MRP1 mRNA.

## 1. Introduction

Both smoking and ambient airborne particulate matter (PM) with an aerodynamic diameter less than 2.5 *μ*m (PM2.5) are capable of inducing noxious particles or gases in the lung [1], which are the principal risk factors for chronic obstructive pulmonary disease (COPD). Proteins of the ATP-binding cassette (ABC) superfamily, such as the multidrug resistance-associated protein 1 (MRP1), play an important role in normal physiology by protecting tissues from toxic xenobiotics and endogenous metabolites [2, 3]. In normal lung tissue, the protein level of MRP1 is high in bronchial epithelium. However, bronchial biopsies of COPD patients indicated that the protein expression of MRP1 was lower in patients with COPD than healthy humans [4]. In our previous study, the results showed that the protein expression of the bronchial epithelial MRP1 was significantly decreased in papain and smoking induced COPD rat model [5]. So far, MRP1 is known as an essential factor for maintaining tissue homeostasis to defend certain tissues against the stress from xenobiotic insults, proinflammatory cysteinyl, and a vast array of other endo- and xenobiotic organic anions [6].

Several signaling pathways, such as mitogen activated protein kinase (MAPK) and phosphatidylinositide 3-kinase/serine-threonine kinase (PI3K/Akt), were reported to regulate the expression of MRP1 [7–9]. Earlier studies indicated that the classical MAPK pathways play an important role in regulating gene expression in eukaryotic cells and they link extracellular signals to the machinery that controls fundamental cellular processes [10, 11]. Among these pathways, c-Jun NH2-terminal kinase (JNK) and extracellular signal-regulated kinase (ERK) were found to be involved in MRP1-mediated multidrug resistance of malignant tumor cells [7, 12]. In addition, PI3K/Akt signaling pathway is another important intracellular signaling pathway which is also involved in the drug resistance of different types of human neoplasm cells [13, 14]. LY294002, a PI3K-specific inhibitor, has been reported to reduce the activity of the MRP1 promoter by stimulating vascular endothelial growth factor (VEGF) [15]. In addition, treatment of K562 cell line with LY294002 or Akt siRNA downregulated P-glycoprotein (P-gp) and MRP1 expression [9]. Although these studies are informative and suggesting a possible relationship between these signaling pathways and MRP1 expression, the mechanism between these three pathways and MRP1 expression in human bronchial epithelial cell line needs to be further studied.

The compounds isothiocyanates (ITCs) from many cruciferous vegetables like cabbage and cauliflower were reported to be useful for treating COPD. Many ITCs have been demonstrated to have chemopreventive activity in a number of inflammatory disorders and modulation of cellular redox status [16–18]. Up to now, most studies were focused on the effect that isothiocyanates can reverse multidrug resistance mediated by MRP1 in cancer cells [19, 20]. However, little is known regarding the effect of isothiocyanates on the expression of MRP1 in normal lung tissue. Allyl isothiocyanate (AITC), a structurally related compound of isothiocyanates, can improve the lung function of COPD rats which is induced by cigarette smoke [21]. Our previous study found that AITC can increase the expression and activity of MRP1 in 16HBE14o- cells [22]. However, the mechanisms remain unknown. Therefore, in this study, we tried to elucidate the contribution of the ERK1/2, JNK, and PI3K/Akt signaling pathways to AITC upregulation of MRP1 activity and expression in 16HBE14o- cells.

## 2. Materials and Methods

### 2.1. Reagents

AITC was purchased from Anhui Haibei Import and Export Company (Hefei, Anhui, China). RPMI 1640 medium and Fetal Bovine Serum were purchased from Gibco. LY294002, U0126, SP600125, 5-CFDA, and sodium dodecyl sulfate (SDS) and dimethyl sulphoxide (DMSO) were purchased from Sigma-Aldrich (Oakville, ON, Canada). Monoclonal antibodies, including anti-MRP1, *β*-actin, and JNK, were purchased from Santa Cruz Biotechnology (Santa Cruz, CA, USA). p-JNK antibody was purchased from Cell Signaling Technology (Danvers, MA, USA). Trizol reagent was purchased from Invitrogen (Carlsbad, CA, USA).

### 2.2. Cell Culture and Treatments

The human bronchial epithelial cell line 16HBE14o- was purchased from Shanghai Fuxiang Biological Technology Company (Shanghai, China). Cells were grown in RPMI-1640 medium supplemented with 10% FBS. Cells were maintained at 37°C in 5% CO_2_ atmosphere. Cells were routinely subcultured prior to reaching 80% confluence. AITC was dissolved in DMSO. The previous study showed that AITC (40 *μ*M) had no significant effect on cell viability of 16HBE14o- after incubating for 24 h [22]. Therefore, the 16HBE14o- cells were cultured in serum-free medium with AITC (40 *μ*M) for 24 h. In the meantime, control cells were treated with 0.1% DMSO only. To explore possible signaling pathways, 16HBE14o- cells were preincubated with either ERK kinase inhibitor U0126 (20 *μ*M) or JNK inhibitor SP600125 (20 *μ*M) or PI3K/Akt inhibitor LY294002 (10 *μ*M) for 30 min and 60 min before the addition of AITC.

### 2.3. RNA Extraction and Real-Time Polymerase Chain Reaction (RT-PCR)

Total RNA was isolated from 16HBE14o- cells, by using TRIzol reagent (Invitrogen, Carlsbad, CA), following the manufacturer's instructions. The concentrations and A260/A280 ratios of the isolated RNAs were determined from the absorbance at 260 and 280 nm by a Hitachi spectrophotometer (modelU1100) and the integrity was verified by agarose gel electrophoresis. cDNA was generated using the High-Capacity cDNA Archive Kit according to the manufacturer's instructions. Real-time PCR was performed using the SYBR Green Master Mix system (Applied Biosystems, CA, USA) according to the manufacturer's instructions on an ABI 7500 real-time PCR machine (Applied Biosystems). Following the reverse transcription reaction, 2 *μ*L of the resultant cDNA was used for PCR amplification reaction as follows: 10 min at 95°C, followed by 40 cycles of 15 s at 95°C and 1 min at 60°C. Primers pairs for each transcript were MRP1 forward 5′-CCTGGAGCTGGCCCACCTGA-3′ and reverse 5′-CGCTGCCCGACACTGAGGTT-3′. GAPDH was used as housekeeping gene, forward 5′-CAAGGCTGTGGGCAA-GGT-3′ and reverse 5′-GGAAGGCCATGCCAGTGA-3′.

### 2.4. Western Blotting Analysis

16HBE14o- cells were plated in 6-well plates and, after 24 h, the growth media were replaced with free-serum and grown overnight. Cells harvested after treatment with inhibitor or AITC were gently washed with ice-cold PBS, and they were placed on ice for 10 min and suspended in 1x cell lysis buffer (Invitrogen), and it was supplemented with 2 mM phenylmethanesulfonyl fluoride (PMSF) and a proteinase inhibitor mixture. The protein concentration of the supernatant was measured using BCA reagents (Pierce, Rockford, IL). Proteins were separated by running through 6% SDS-PAGE gel and transferred to a polyvinylidene difluoride membrane. After being blocked with 5% nonfatted milk, the membrane was probed by specific antibodies for 1 h at 37°C and 4°C overnight and the bands were visualized using the ECL Plus kit according to the manufacturer's instructions. Kodak 1D image analysis software was used to analyse the Western blotting results.

### 2.5. Flow Cytometry

The impact of JNK pathway on MRP1 activity was examined using a 5-CFDA efflux assay in 16HBE14o- cells with AITC. When 5-CFDA diffuses freely into cells, it is converted to carboxyfluorescein (CF) which is a substrate of MRP1 for efflux. Confluent cells were preloaded with 1 *μ*M 5-CFDA for 1 h. The cells were incubated with AITC (40 *μ*M) for 24 h after pretreatment with or without the addition of SP600125 (20 *μ*M) for 60 min. After incubation, cells were detached and centrifuged at 500 ×g for 5 min, and the pellets were suspended in 1 mL of ice-cold PBS and immediately placed on ice. Intracellular 5-carboxyfluorescein (5-CF) fluorescence intensity was measured using flow cytometry for 30 min using an ABI LSRII flow cytometer (Applied Biosystems). We measured 10,000 events per sample (living cells). Samples were excited at 488 nm using an argon laser, and the emission fluorescence was detected at 530 nm. The Winlist 5.1 program (Verity Software House Inc., Topsham, ME, USA) was used to calculate the mean fluorescence intensity (MFI) values.

### 2.6. Statistical

Analysis Student's *t*-test and one-way ANOVA were used to calculate significant differences. Differences were considered significant when *P* < 0.05. Statistical analyses were performed with SPSS 17.0 (SPSS Inc., Chicago, IL).

## 3. Results

### 3.1. AITC Induced MRP1 Gene Expression via JNK but Not ERK or PI3K/Akt Pathways

**Figure 1:**
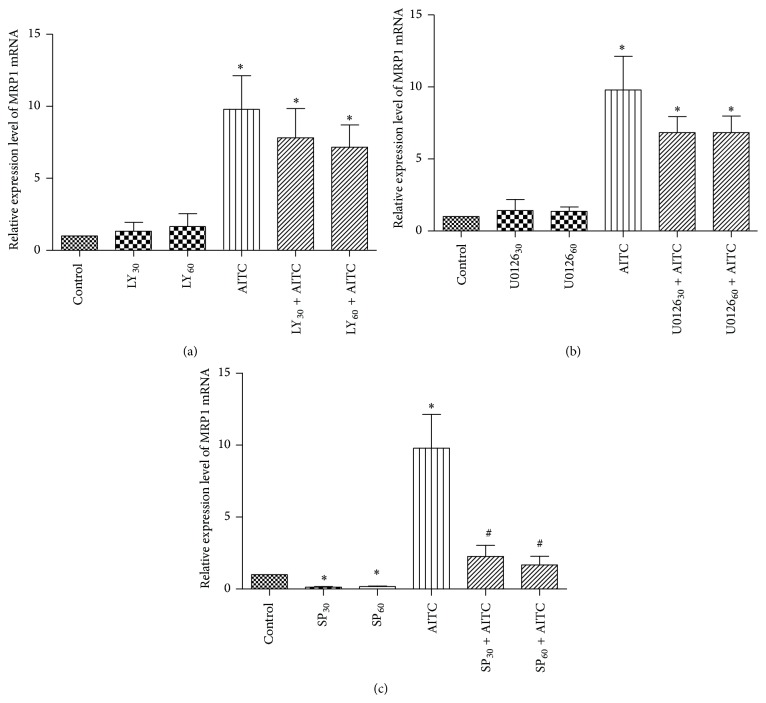
Effect of cell signaling pathway inhibitor on MRP1 mRNA expression in 16HBE14o- cells. (a) Cells were pretreated with or without PI3K/Akt inhibitor LY294002 (10 *μ*M) 30 min or 60 min prior to the exposure to AITC for 24 h, and then the relative expression level of MRP1 was measured by RT-PCR. (b) Cells were pretreated with or without ERK inhibitor U0126 (20 *μ*M) 30 min or 60 min prior to the exposure to AITC for 24 h, and then the relative expression level of MRP1 was measured by RT-PCR. (c) Cells were pretreated with or without JNK inhibitor SP600125 (20 *μ*M) for 30 min or 60 min prior to the exposure to AITC for 24 h, and then the relative expression level of MRP1 was measured by RT-PCR.  ^*∗*^Significant difference from control, *P* < 0.05.  ^#^Significant difference from corresponding AITC control, *P* < 0.05.

To reveal the mechanism of MRP1 mRNA expression induced by AITC, 16HBE14o- cells were pretreated with JNK, ERK, and PI3K/Akt inhibitors for 30 min or 60 min prior to the exposure to AITC for 24 h. As shown in Figures 1(a) and 1(b), MRP1 mRNA expression in 16HBE14o- cells was not obviously affected by application of ERK inhibitor U0126 (20 *μ*M) or PI3K/Akt inhibitor LY294002 (10 *μ*M) for 30 min or 60 min. However, incubation of bronchial epithelial cells with JNK inhibitor SP600125 (20 *μ*M) significantly attenuated MRP1 mRNA level (Figure 1(c)). To further examine whether these pathways are involved in AITC-induced MRP1 expression, we observed the effect of specific inhibitors of PI3K/Akt, ERK, and JNK on AITC-induced MRP1 mRNA expression. AITC significantly increased MRP1 mRNA expression (Figures 1(a)–1(c)); Table 1. AITC-induced expression was significantly depressed by SP600125 (20 *μ*M) (Figure 1(c)). In contrast, U0126 (20 *μ*M) and inhibitor LY294002 (10 *μ*M) produced little effect on AITC-induced MRP1 mRNA expression (Figures 1(a) and 1(b)). Together, these results indicated that JNK but not ERK or PI3K/Akt pathway contributes to AITC-induced MRP1 mRNA expression.

### 3.2. AITC Induced the Protein Expression of MRP1 via JNK Signaling Pathway

We then examined whether AITC affected MRP1 activity due to the upregulated MRP1 mRNA expression following JNK pathway activation. 16HBE14o- cells were pretreated with SP600125 for 60 min before treatment with or without AITC (40 *μ*M) for 24 h. The phosphorylation status of JNK was measured using antibody that specifically recognizes the phosphorylated form of JNK. The ratio of phosphorylated JNK to total JNK (p-JNK/t-JNK) was used to evaluate the activity of JNK.

**Figure 2:**
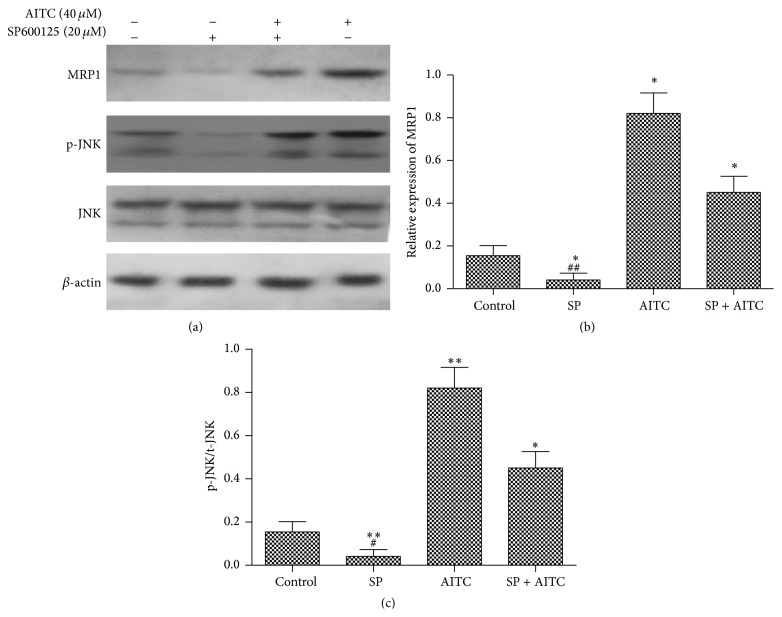
JNK signaling pathway is required for the expression of MRP1 induced by AITC. (a) 16HBE14o- cells were pretreated with SP600125 for 60 min before presence or absence of 40 *μ*M AITC for 24 h, and then the expression of MRP1 protein was measured by Western blot. Densitometric analysis of data from 16HBE14o- cells shows the effects of AITC and/or JNK on MRP1 levels (b) and relative expression of p-JNK/t-JNK (c). Data was normalized against control and presented as the mean ± S.D. Experiments were performed in triplicate.  ^*∗*^Significant difference from control, *P* < 0.05.  ^*∗∗*^Significant difference from control, *P* < 0.01.  ^#^Significant difference from corresponding controls (SP + AITC), *P* < 0.05.  ^##^Significant difference from corresponding controls (SP + AITC), *P* < 0.01.

SP600125 alone significantly decreased the expression of MRP1 protein in 16HBE14o- cells (Figure 2(b)). In contrast, AITC (40 *μ*M) significantly increased MRP1 protein and the increase was remarkably inhibited by pretreatment with SP600125 (Figure 2(b)). In the meantime, AITC (40 *μ*M), but not the vehicle control (DMSO) or JNK inhibitor, significantly increased JNK phosphorylation and the ratio of p-JNK/t-JNK (Figure 2(c)). Pretreatment of SP600125 (20 *μ*M) inhibited the upregulation of p-JNK/t-JNK ratio by AITC. These results suggest that activated JNK signaling pathway contributes to the upregulation of MRP1 protein by AITC, supporting the functional role of increased MRP1 mRNA expression.

### 3.3. Effect of JNK Inhibitor on AITC-Induced MRP1 Function in 16HBE14o- Cells

**Figure 3:**
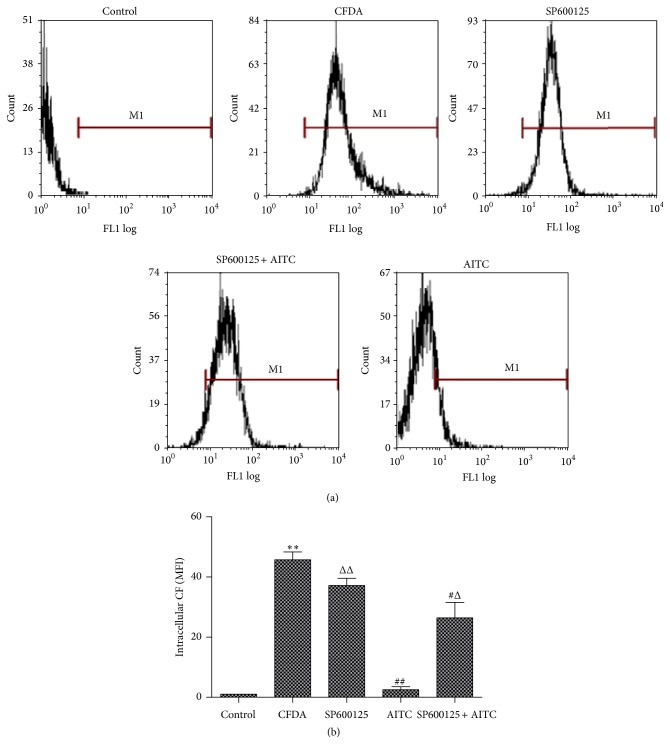
Effect of JNK cell signaling pathway inhibitor on MRP1 function in 16HBE14o- cells. (a) Cells were pretreated with or without the addition of SP600125 (20 *μ*M) for 60 min and then incubated with AITC (40 *μ*M) for 24 h and the fluorescence of the CF retained within the cells was evaluated by flow cytometry. (b) Intracellular CF (MFI) retention as a measure of MRP1 function in 16HBE14o- cells.  ^*∗∗*^Significant difference from control, *P* < 0.01.  ^#^Significant difference from CFDA, *P* < 0.05.  ^##^Significant difference from CFDA, *P* < 0.01.  ^Δ^Significant difference from AITC alone, *P* < 0.05.  ^ΔΔ^Significant difference from AITC alone, *P* < 0.01.

To determine the role of JNK signaling pathway on AITC-induced MRP1 function, 16HBE14o- cells were treated with JNK inhibitor SP600125 (20 *μ*M). 5-CFDA was always used as a model MRP1 substrate to evaluate the function of MRP1 [23].  Figure 3(a) depicts the intracellular fluorescence of CF which was measured with flow cell cytometry when cells were incubated with AITC (40 *μ*M) with or without pretreatment of SP600125 (20 *μ*M). Intracellular fluorescence was significantly increased when cells were loaded with 5-CFDA (Figure 3(a)), suggesting that 5-CFDA were effectively permeated to cells. AITC treatment significantly decreased intracellular CF fluorescence compared to 5-CFDA treatment alone (Figure 3(b)), suggesting a higher function of MRP1. However, the AITC-induced decrease of intracellular CF fluorescence was significantly antagonized by pretreatment of SP600125 (20 *μ*M) (Figure 3(b)), indicating that AITC-induced increase of MRP1 function was inhibited by SP600125. These results confirmed that the pharmacological blockade of JNK signal pathway plays an important role in the regulation of AITC-induced MRP1 function.

## 4. Discussion

ABC transporters (i.e., MRP1,P-gp), as an ATP-dependent pump, act as a gatekeeper against numerous xenobiotics, secretion of toxic compounds, apoptosis, and the immune response [24, 25]. MRP1 is also associated with lung function and inflammatory markers in COPD patients [26]. The clinically used pulmonary drugs such as budesonide, formoterol, and ipratropium bromide are very likely to affect MRP1 activity, besides their positive effects on respiratory symptoms [2]. Some endogenous substances such as the proinflammatory leukotriene C(4) and antioxidant glutathione are known substrates for MRP1 and have a close relationship with COPD [27–29]. Therefore, to better understand the mechanism of COPD, it is important to know how the MRP1 expression is regulated.* In vitro* and* in vivo* studies have shown that AITC upregulated the expression and function of MRP1 [22]. Our present findings, for the first time, indicate that JNK MAP kinase pathway contributes to AITC-induced MRP1 expression in human bronchial epithelial cell.

Multiple mechanisms were reported to be involved in the cellular response to AITC. Previous studies indicated that PEITC and/or its conjugates are MRP1 substrates, suggesting that binding interactions with the unmodified molecules was involved in MRP1 inhibition [30]. However, the underlying mechanisms for the function and expression of MRP1 is still unclear. Recently, accumulating evidences suggest that the activation of ERK, JNK, or PI3K/Akt signaling pathway may play an important role in chemoresistance of several cancers cells [7, 12, 31]. U0126 (3–20 *μ*M), LY294002 (5–10 *μ*M), and SP600125 (10–20 *μ*M) were found to selectively inhibited ERK, PI3K/Akt, and JNK pathway, respectively [9, 32, 33]. In this study, we thus used the selective inhibitors to examine the effect of the activation of ERK, JNK, and PI3K/Akt pathways on AITC-induced upregulation of MRP1 expression. Our results indicated that JNK but not ERK or PI3K/Akt pathway plays an important role in MRP1 mRNA expression. In addition, AITC increased MRP1 mRNA expression in 16HBE14o- cells. Inhibition of JNK signaling pathway by selective JNK pathway inhibitor SP600125 abolished AITC-induced upregulation of MRP1 mRNA level. However, ERK and PI3K/Akt pathways produced no obvious effect. We also tested the effect of JNK pathway on phosphorylated JNK based on the result that JNK pathway regulated AITC-induced MRP1 mRNA. In contrast to the inhibition of SP600125 on JNK pathway activation, AITC reversed SP600125-inhibition on the activity of JNK signaling which is important in regulating cell apoptosis and stress responses [34]. As MRP1 is a protective protein for protecting tissues from toxic xenobiotics and endogenous metabolites [2, 3], its expression and activity are critical for COPD. AITC decreased CF accumulation by upregulating MRP1 function in 16HBE14o- cells. However, AITC-induced decrease of CF was reversed by SP600125. Based on the results, we could reasonably make the conclusion that AITC upregulation of MRP1 is JNK pathway-dependent. JNK signaling pathway was reported to be required for MRP1 expression by another compound which has been reported in other cell systems [12]. Tang et al. reported that phenethyl isothiocyanate, one of the isothiocyanates, was found to downregulated multidrugs resistance 1 and MRP1 expression through blocking Akt and activating JNK pathway in T24/Adriamycin cells [35]. Therefore, our present study for the first time confirmed that AITC increase MRP1 expression in human bronchial epithelial cells through activation of JNK pathway. Previous study indicated that PI3K/Akt signaling pathway participated in regulating MRP1 expression by isothiocyanates [20]. However, our results showed no obvious effect of AITC on MRP1 expression in 16HBE14o- cells.

Smoking is not the only part factor of smokers developing COPD, but it is the main principal risk factor [24]. However, little is known about* in vivo* detoxification and elimination processes of noxious substances after cigarette smoke is absorbed in lung. During the development of COPD, cigarette smoke extract was reported to affect the protective activity of MRP1 on lung tissue [24]. Additional studies reported that JNK expression in lung parenchyma was increased after tobacco smoke exposure for 5 days in rats and 4–12 weeks in guinea pigs [36, 37]. In contrast, upregulation of phosphorylated keratin type 2 cytoskeletal 8 (K8) and keratin type 1 cytoskeletal 18 (K18) is to moderate JNK signaling in lung tissue of rats after a short time of tobacco smoke exposure [38]. Taking all into consideration, what happens to the activity of JNK and the downstream events in response to tobacco smoke and/or pretreatment with AITC is not clear. Therefore, it is important to study the regulation mechanism about AITC-regulated MRP1 in stimulation with cigarette smoke extract.

In summary, our study indicated that AITC enhanced the expression of MRP1 at both mRNA level and protein level via JNK signaling pathway but not PI3K/Akt or ERK pathway in 16HBE14o- cells. Understanding the mechanisms of MRP1 regulated by AITC may help us to produce a new therapeutic approach for the reversal and prevention of COPD.

## Figures and Tables

**Table 1:** Effect of cell signaling pathway inhibitor on MRP1 *mRNA* expression.

	GAPDH	MRP1	ΔCt	ΔΔCt	RQ = 2^−ΔΔCt^
Control	15.12 ± 0.52	19.72 ± 0.50	4.60 ± 0.41	0	1
AITC	14.96 ± 0.67	16.30 ± 0.58	1.34 ± 0.17	−3.27 ± 0.34	9.79 ± 2.34^*∗*^
LY_30_	15.23 ± 0.45	19.53 ± 0.94	4.31 ± 1.03	−0.29 ± 0.72	1.33 ± 0.62
LY_30_ + AITC	14.85 ± 0.66	16.54 ± 0.49	1.68 ± 0.20	−2.92 ± 0.38	7.73 ± 2.13^*∗*^
LY_60_	14.97 ± 0.71	19.00 ± 1.36	4.02 ± 1.16	−0.58 ± 0.87	1.67 ± 0.90
LY_60_ + AITC	15.36 ± 0.76	17.51 ± 0.96	1.79 ± 0.26	−2.81 ± 0.32	7.16 ± 1.55^*∗*^
U_30_	14.79 ± 0.24	19.03 ± 1.39	4.24 ± 1.15	−0.36 ± 0.74	1.41 ± 0.78
U_30_ + AITC	14.83 ± 0.54	16.67 ± 1.05	1.84 ± 0.59	−2.76 ± 0.25	6.83 ± 1.11^*∗*^
U_60_	14.74 ± 0.70	18.92 ± 1.02	4.18 ± 0.64	−0.42 ± 0.35	1.37 ± 0.31
U_60_ + AITC	14.95 ± 0.62	16.79 ± 0.65	1.87 ± 0.37	−2.76 ± 0.23	6.84 ± 1.15^*∗*^
SP_30_	15.58 ± 0.18	23.05 ± 0.70	7.47 ± 0.79	2.87 ± 0.42	0.14 ± 0.04^*∗*^
SP_30_ + AITC	15.45 ± 0.66	19.13 ± 0.58	3.68 ± 0.51	−0.92 ± 0.17	2.26 ± 0.78^#^
SP_60_	15.56 ± 0.45	22.71 ± 0.68	7.15 ± 0.30	2.54 ± 0.35	0.18 ± 0.04^*∗*^
SP_60_ + AITC	15.12 ± 0.61	19.04 ± 0.39	3.92 ± 0.81	−0.68 ± 0.55	1.68 ± 0.60^#^

Data were normalized against control and presented mean ± S.D. Experiments were performed in triplicate.

^*∗*^Significant difference from control, *P* < 0.05.

^#^Significant difference from corresponding AITC control, *P* < 0.05.
